# Hydro­thermal synthesis and crystal structure of poly[bis­(μ_3_-3,4-di­amino­benzoato)manganese], a layered coordination polymer

**DOI:** 10.1107/S2056989020006805

**Published:** 2020-05-22

**Authors:** Muhammad Kaleem Khosa, Paul T. Wood, Simon M. Humphrey, William T. A. Harrison

**Affiliations:** a Department of Chemistry, Government College University, Faisalabad 38000, Pakistan; b Department of Chemistry, University of Cambridge, Lensfield Road, Cambridge CB2 1EW, England; c Department of Chemistry and Biochemistry, The University of Texas at Austin, 1 University Station, Austin, Texas 78712, USA; d Department of Chemistry, University of Aberdeen, Meston Walk, Aberdeen AB24 3UE, Scotland

**Keywords:** manganese, ligand, layered structure, coordination polymer, crystal structure

## Abstract

The hydro­thermal synthesis and crystal structure of the title two-dimensional coordination polymer, poly[bis­(μ_3_-3,4-di­amino­benzoato-κ^3^ *N* ^3^,*O*,*O*′)manganese(II)], [Mn(C_7_H_7_N_2_O_2_)_2_]_*n*_, are described. The Mn^2+^ cation (site symmetry ) adopts a tetra­gonally elongated *trans*-MnN_2_O_4_ octa­hedral coordination geometry and the μ^3^-*N*,*O*,*O*′ ligand (bonding from both carboxyl­ate O atoms and the *meta*-N atom) links the metal ions into infinite (10) layers. The packing is consolidated by intra-layer N—H⋯O and inter-layer N—H⋯N hydrogen bonds. The structure of the title compound is compared with other complexes containing the C_7_H_7_N_2_O_2_^−^ anion and those of the related *M*(C_8_H_8_NO_2_)_2_ (*M* = Mn, Co, Ni, Zn) family, where C_8_H_8_NO_2_^−^ is the 3-amino-4-methyl­benzoate anion.

## Abstract 2

The title compound is a two-dimensional coordination polymer with inter-layer connectivity provided by N—H⋯N hydrogen bonds.

## Chemical context   

The benzoate anion, C_7_H_5_O_2_^−^, is a classic ligand in coordin­ation chemistry, with over 1500 crystal structures reported in the Cambridge Structural Database (Groom *et al.*, 2016 ▸) for complexes of first-row transition metals, which include monodentate (κ*O*), chelating (κ^2^ *O*,*O*′) and bridging (μ^2^-*O*,*O*′) modes for the ligand [for ligand bonding-mode notation, see Janicki *et al.* (2017 ▸)]. Functionalized benzoate derivatives add further structural variety: for example, –NH_2_ substituents at the *ortho*, *meta* and/or *para* positions of the benzene ring can form or accept hydrogen bonds with respect to nearby acceptor or donor groups and/or bond to another metal ion (*i.e.*, as a possible μ^2^-*N*,*O* or μ^3^-*N*,*O*,*O*′ bridging ligand).
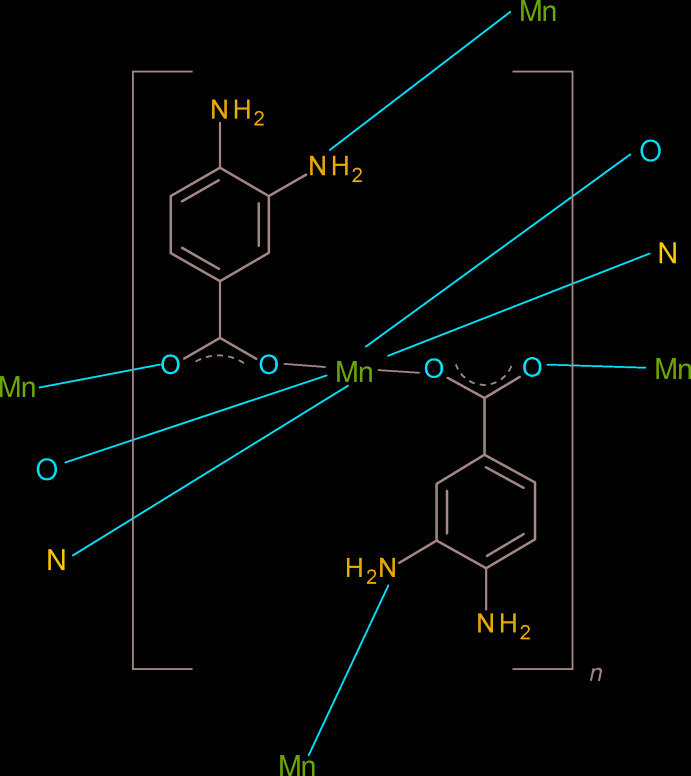


As part of our ongoing studies in this area (Khosa *et al.*, 2015 ▸), we now describe the hydro­thermal synthesis and crystal structure of the title compound (I), where C_7_H_7_N_2_O_2_^−^ is the 3,4-di­amino­benzoate (dbz^−^) anion.

## Structural commentary   

**Figure 1:**
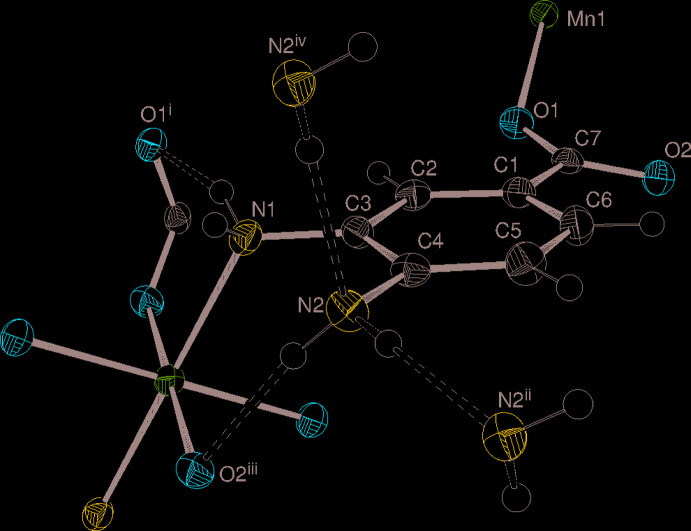
Fragment of the structure of (I) showing 50% displacement ellipsoids emphasizing the μ^3^-*N*,*O*,*O*′ ligand bonding mode and displaying hydrogen bonds as double-dashed lines. [Symmetry codes as in Table 1 ▸; additionally: (iv) −*x* + 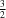, *y* + 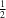, −*z* + 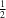.]

The title complex (I) consists of an Mn^2+^ cation located on a crystallographic inversion centre and one deprotonated dbz^−^ ligand with its atoms lying on general positions (Fig. 1 ▸), which of course generates the overall 1:2 metal to ligand ratio and ensures charge balance. The C7/O1/O2 carboxyl­ate group of the ligand is rotated from the plane of the C1–C6 aromatic ring by 13.5 (2)° and the C—O bonds [C7—O1 = 1.261 (3) Å and C7—O2 = 1.277 (3) Å] are of similar length, indicating substantial electronic delocalization. The C2—C3 and C5—C6 bonds (mean = 1.383 Å) are marginally shorter than the other bonds in the benzene ring (mean = 1.396 Å), which can be related to resonance of the *para*-N atom lone pair with the carboxyl­ate group (Mukombiwa & Harrison, 2020 ▸). This is also presumably reflected in the fact that the C4—N2 bond length [1.413 (3) Å] is slightly shorter than C3—N1 [1.432 (3) Å]. Even so, it is noteworthy that the bond-angle sums about atoms N1 and N2 of 329.8 and 335.8°, respectively, are indicative of a significant tendency towards *sp* ^3^ hybridization of the N atoms, *i.e.*, localization of the lone pairs. In terms of the non-hydrogen atoms attached to the benzene ring, N1 and N2 deviate slightly from the mean plane of the ring in opposite directions by −0.023 (3) and 0.024 (3) Å, respectively, whereas atom C7 shows a larger deviation of −0.077 (3) Å.

The dbz^−^ ligand bonds to three different metal ions from both of its carboxyl­ate O atoms and also from its *meta*-N atom (Fig. 1 ▸), *i.e.*, μ^3^-*N*,*O*,*O*′ mode. The preference for the *meta-*N atom to bond to the metal ion (rather than the *para-*N atom) can be related to the resonance effect noted in the previous paragraph. With respect to the carboxyl­ate group, the metal ion bonded to O1 is displaced in an ‘upwards’ sense by 1.046 (7) Å and the other (bonded to O2) is displaced ‘downwards’ by −1.651 (6) Å.

Crystal symmetry generates a centrosymmetric *trans*-MnN_2_O_4_ elongated octa­hedron for the metal ion, in which the Mn1—N1 bond length of 2.3065 (19) Å is distinctly longer than the Mn1—O1 [2.1591 (14) Å] and Mn1—O2 [2.2062 (15) Å] bonds. The manganese ion presumably has a high-spin 3*d* ^5^ configuration, thus the distortion of the octa­hedron cannot be electronic in nature (*i.e.*: a Jahn–Teller effect) and might arise for steric reasons. The angular variance (Robinson *et al.*, 1971 ▸) of the *cis X*—Mn—*Y* (*X*, *Y* = N, O) bond angles is (7.7°)^2^, indicating relatively little angular distortion from the ideal values of 90°; the minimum and maximum angles are 86.75 (6) and 93.25 (6)°, respectively. The *trans* bond angles are constrained by symmetry to be 180°. The bond-valence sum (in valence units) (Brown & Altermatt, 1985 ▸) for the metal ion is 1.97, in very good agreement with the expected value of 2.00 for Mn^2+^.

**Figure 2:**
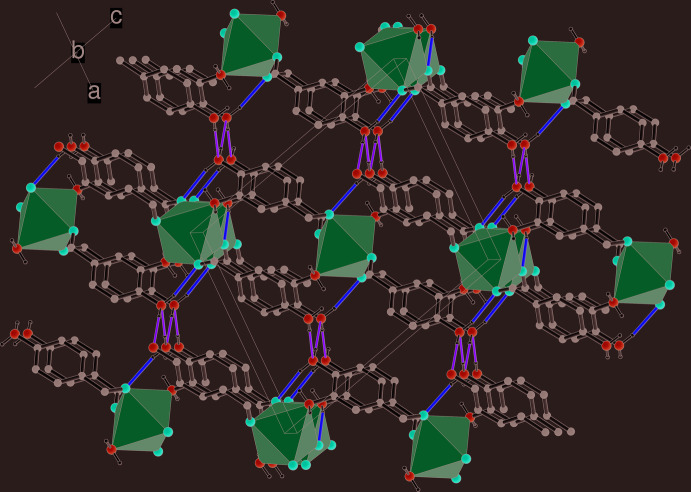
Packing diagram for (I) viewed down [010], showing the (10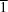) layers (seen edge-on) with the MnN_2_O_4_ octa­hedra shown in polyhedral representation and intra­layer N—H⋯O and inter­layer N—H⋯N hydrogen bonds shown as yellow and green lines, respectively.

In the extended structure of (I), the μ^3^ bridging ligand links the metal ions into infinite (10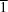) sheets (Fig. 2 ▸). These sheets can be decomposed into [010] chains of octa­hedra linked by the bridging C7/O1/O2 carboxyl­ate groups [shortest Mn⋯Mn(*x*, *y* + 1, *z*) separation = 4.4212 (2) Å], with connectivity in the [101] direction achieved *via* the benzene ring of the ligands and their *meta*-N atoms [shortest Mn⋯Mn(−*x* + 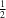, *y* + 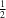, −*z* + 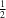) = 8.1520 (4) Å].

Hydrogen bonding helps to consolidate the structure of (I): the *para* –N2H_2_ group forms an intra-sheet N2—H4*N*⋯O2^iii^ bond (Fig. 1 ▸ and Table 1 ▸, where symmetry codes are defined) but also participates in an *inter*-sheet N2—H3*N*⋯N2^ii^ link, *i.e.*, N2 ‘accepts its own hydrogen bond’ from an adjacent symmetry related –N2H_2_ group and *C*(2) infinite chains propagating in the [010] direction arise in the crystal (Figs. 1 ▸ and 2 ▸) with adjacent N atoms related by the 2_1_ screw axis; it is notable that the –NH_2_ groups in adjacent layers are aligned opposite to each other to facilitate the formation of this inter-sheet hydrogen bond. As well as forming a coordinate bond to the metal ion from N1, the *meta* –N1H_2_ group forms an N1—H1*N*⋯O1^i^ intra-sheet hydrogen bond while the N1—H2*N* group does not participate in a hydrogen bond, perhaps due to steric crowding. There are no significant aromatic π–π stacking inter­actions in the crystal of (I), the shortest centroid–centroid separation between C1–C6 rings being 4.4211 (13) Å.

## Hirshfeld surface analysis   

**Figure 3:**
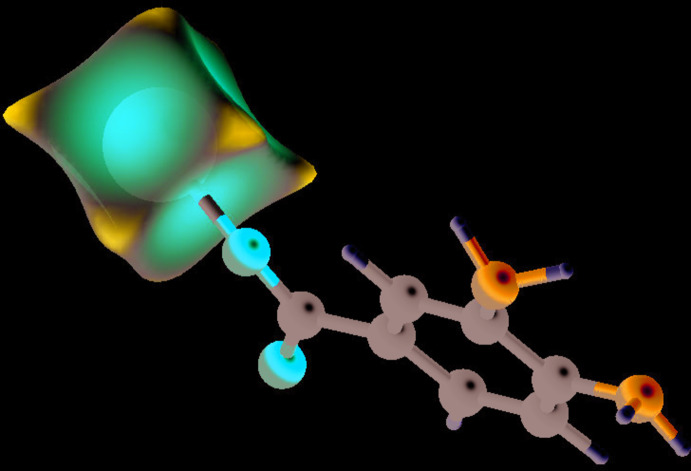
Hirshfeld surface mapped on *d*_norm_ for the Mn^2+^ cation in (I).

In order to gain further insight into non-covalent inter­actions in the crystal of (I), the Hirshfeld surface and two-dimensional fingerprint plots were calculated using *CrystalExplorer* (Turner *et al.*, 2017 ▸) following the approach recently described by Tan *et al.* (2019 ▸). The Hirshfeld surface of the dbz^−^anion in (I) (see supporting information) largely shows the expected red spots of varying intensity corresponding to close contacts resulting from the O—Mn and N—Mn coordinate bonds and N—H⋯O and N—H⋯N hydrogen bonds described above. The Hirshfeld surface mapped onto *d*_norm_ for the manganese cation in (I) (Fig. 3 ▸) is a distinctive ‘dimpled cube’, with the intense red spots (short inter­actions) corresponding to its coordinate bonds, which correlates nicely with its octa­hedral coordination geometry.

The most important outward (*i.e.*, non-reciprocal) percentage contributions of the different type of contacts for the anion and the cation are listed in Table 2 ▸. It may be seen that H⋯H (van der Waals) contacts are by far the most significant contributor for the anion followed by C⋯H and H⋯C contacts (total contribution = 59.9%). The contacts associated with the hydrogen bonds, *i.e.*, H⋯N (donor), H⋯O (donor), N⋯H (acceptor) and O⋯H (acceptor) collectively account for some 24.2% of the surface. Finally, the coordinate bonds to the metal ion (O—Mn and N—Mn), despite their presumed importance in establishing the crystal structure, account for a modest 7.2% of the anion’s surface.

As might be expected, the Mn—O contacts (62.0%) for the cation dominate its Hirshfeld surface, followed by Mn—N (19.3%), but the ratio of these (∼3.2:1) deviates significantly from the simple 2:1 ratio that would reflect the four Mn—O bonds and two Mn—N bonds. The high percentage of Mn⋯H contacts (18.8%) is notable: these occur at the corners of its cube-like Hirshfeld surface (Fig. 3 ▸) and seem to arise from the proximity to the metal ion of the two H atoms attached to N1 [Mn1⋯H1*N* = 2.58 (3); Mn1⋯H2*N* = 2.68 (3) Å] and might be repulsive in nature. For further discussion of short metal⋯hydrogen contacts in coordination compounds and their Hirshfeld surfaces, see Pinto *et al.* (2019 ▸).

**Figure 4:**
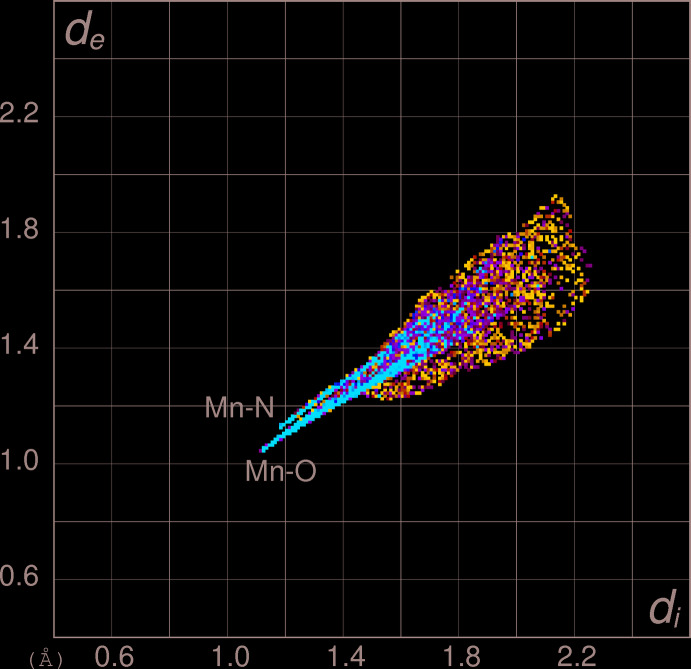
Two-dimensional Hirshfeld fingerprint plot for the Mn^2+^ cation in (I).

The wing-like two-dimensional fingerprint plot for the manganese cation in (I) (Fig. 4 ▸) shows two prominent features: the spike ending at (*d*_i_, *d*_e_) = (∼1.14, ∼1.06 Å) and extending backwards corresponds to the Mn—O coordinate bonds and the (1.18, 1.14 Å) feature just separated from it equates with the Mn—N bonds. The Mn⋯H contacts are overlapped with the Mn—O and Mn—N contacts in the main body of the ‘wing’ with the shortest Mn⋯H contact at about (1.40, 1.25 Å), which correlates well with the short Mn⋯H contacts noted in the previous paragraph.

## Database survey   

**Figure 5:**
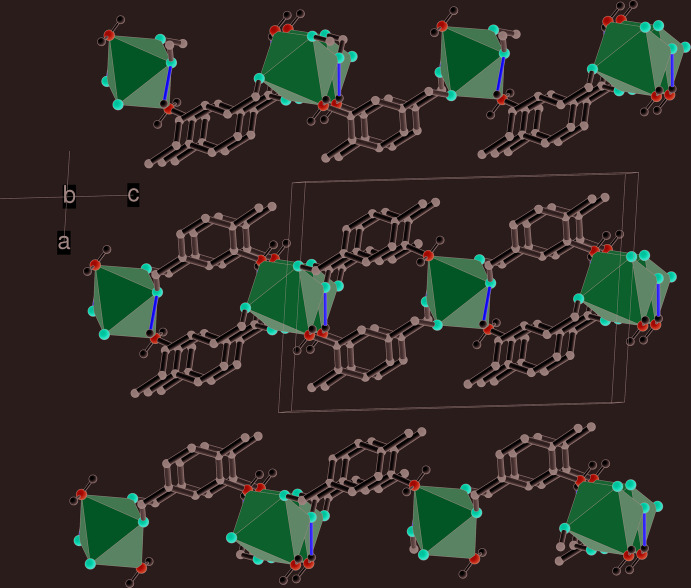
Packing diagram (redrawn from Khosa *et al.*, 2015 ▸) for (II) viewed down [010], with the MnN_2_O_4_ octa­hedra shown in polyhedral representation and N—H⋯*X* hydrogen bonds shown as yellow lines. Note how the layers, which propagate parallel to the (100) plane in this setting of the space group, are laterally shifted compared to those in Fig. 2 ▸: the shortest inter-layer N⋯N separation in (I) is 3.106 (3) Å (*via* the N1—H1*N*⋯N1 hydrogen bond) compared to the shortest inter-layer C⋯C separation of 3.948 (2) Å in (II).

The structure of (I) may firstly be compared with the isostructural *M*(C_8_H_8_NO_2_)_2_ family [*M* = Mn (CCDC refcode ULEZUI) (II), Co (ULIBAU), Ni (ULIBEY) and Zn (ULIBIC)], where C_8_H_8_NO_2_^−^ is the 3-amino-4-methyl­benzoate anion (Khosa *et al.*, 2015 ▸). These phases contain μ^3^-*N*,*O*,*O*′ ligands and centrosymmetric *M*N_2_O_4_ octa­hedra, which generate very similar polymeric layers to those found in (I). In (II), the sheets propagate parallel to the (100) plane of the monoclinic cell due to a different choice of unit-cell setting (see *Refinement* section). The major difference arises with respect to the *para*-substituent: in (II) the methyl groups in adjacent (100) layers are laterally shifted with respect to each other (Fig. 5 ▸) to avoid an unfavourable close steric contact and there are no directional inter-sheet inter­actions beyond normal van der Waals’ contacts, which compares to the inter-layer N—H⋯N links already mentioned for (I).

Despite its potential as a polyfunctional bridging ligand, just five crystal structures of complexes of the 3,4-di­amino­benzoate anion are reported in the Cambridge Structural Database as of March 2020 with sodium (BEHJEE: Rzaczyńska *et al.*, 2003 ▸), zinc (MIWSES; Fernández-Palacio *et al.*, 2014 ▸), tin (XUPRUV and XUPSAC; Pruchnik *et al.*, 2002 ▸) and neodymium (YENKOR; Rzaczyńska *et al.*, 1994 ▸). Key data for (I) and these structures are summarized in Table 3 ▸, which indicates a wide variety of metal coordination polyhedra, ligand bonding modes and topologies. Unlike the isostructural manganese and zinc phases in the *M*(C_8_H_8_NO_2_)_2_ family, MIWSES features a water mol­ecule bonded to the trigonal–bipyramidally coordinated zinc ion and has a quite different overall structure to (I). It may finally be noted that (I) represents the first reported example of a μ^3^-*N*,*O*,*O*′ bonding mode for the dbz^−^ anion.

## Synthesis and crystallization   

A mixture of 99 mg (0.50 mmol) of MnCl_2_·4H_2_O and 152 mg (1.00 mmol) of 3,4-di­amino­benzoic acid were added to 1.0 ml of 1 *M* KOH under stirring. The resulting mixture was heated to 423 K in a 23 ml Teflon-lined autoclave for 10 h. The autoclave was then removed from the oven and cooled to room temperature over several hours and opened. Colourless plates of (I) were recovered by vacuum filtration and rinsed with acetone and dried.

## Refinement   

Crystal data, data collection and structure refinement details are summarized in Table 4 ▸. The N-bound H atoms were located in difference maps and their positions were freely refined. The C-bound H atoms were geometrically placed (C—H = 0.95 Å) and refined as riding atoms. The constraint *U*_iso_(H) = 1.2*U*_eq_(carrier) was applied in all cases. The standard setting of the unit cell for (I) (*i.e.*, space group *P*2_1_/*c*) in which the polymeric layers would propagate in the (100) plane, as also found for compound (II), has *a* = 13.975, *b* = 4.421, *c* = 15.693 Å and β = 134.77°, but *P*2_1_/*n* was chosen here to avoid refinement problems associated with the very obtuse β angle: compare Feast *et al.* (2009 ▸).

## Supplementary Material

Crystal structure: contains datablock(s) I, global. DOI: 10.1107/S2056989020006805/is5538sup1.cif

Structure factors: contains datablock(s) I. DOI: 10.1107/S2056989020006805/is5538Isup2.hkl

Hirshfeld surface of the anion. DOI: 10.1107/S2056989020006805/is5538sup3.docx

CCDC reference: 2004911

Additional supporting information:  crystallographic information; 3D view; checkCIF report

## Figures and Tables

**Table 1:** Hydrogen-bond geometry (Å, °)

*D*—H⋯*A*	*D*—H	H⋯*A*	*D*⋯*A*	*D*—H⋯*A*
N1—H1*N*⋯O1^i^	0.88 (3)	2.19 (3)	3.022 (3)	156 (2)
N2—H3*N*⋯N2^ii^	0.85 (3)	2.26 (3)	3.106 (3)	178 (2)
N2—H4*N*⋯O2^iii^	0.88 (3)	2.15 (3)	3.008 (2)	167 (2)

Symmetry codes: (i) 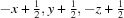; (ii) 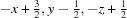; (iii) 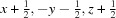.

**Table 2:** Hirshfeld surface contact percentages for (I)

H⋯H	33.6
H⋯C	10.2
H⋯O	8.6
H⋯N	2.5
H⋯Mn	0.9
C⋯H	16.1
C⋯C	3.1
O⋯Mn	5.2
O⋯H	10.6
N⋯H	2.5
N⋯Mn	2.0
Mn⋯O	62.0
Mn⋯N	19.3
Mn⋯H	18.8

All contacts are ‘non-reciprocal’ (*i.e.*, outward facing).

**Table 3:** Structural features of complexes containing the C_7_H_7_N_2_O_2_^−^ anion

Code/refcode	Metal coordination polyhedron	Ligand bonding mode	Topology
(I)	MnN_2_O_6_ octa­hedron	μ^3^-*N*,*O*,*O*	Layered
BEHJEE	NaO_6_ octa­hedron^*a*^	κ*O*	Chain
MIWSES	ZnO_3_N_2_ trigonal bipyramid^*a*^	μ^2^-*N*,*O*	Chain
XUPRUV	SnC_3_O tetra­hedron^*b*^	κ*O*	Mol­ecular
XUPSAC	SnC_3_NO trigonal bipyramid	μ^2^-*N*,*O*	Chain
YENKOR	NdO_9_ capped square anti­prism^*a*^	κ*O* and κ^2^ *O*,*O*′	Mol­ecular

Notes: (*a*) Some of the ligands are water mol­ecules of crystallization; (*b*) a short ‘secondary’ Sn⋯O contact (2.68 Å) is also present.

**Table 4:** Experimental details

Crystal data	
Chemical formula	[Mn(C_7_H_7_N_2_O_2_)_2_]
*M*_r_	357.23
Crystal system, space group	Monoclinic, *P*2_1_/*n*
Temperature (K)	180
*a*, *b*, *c* (Å)	11.5171 (5), 4.4212 (2), 13.9752 (7)
β (°)	104.698 (3)
*V* (Å^3^)	688.32 (6)
*Z*	2
Radiation type	Mo *K*α
μ (mm^−1^)	0.99
Crystal size (mm)	0.23 × 0.10 × 0.05

Data collection	
Diffractometer	Nonius KappaCCD
Absorption correction	Multi-scan (*SORTAV*; Blessing, 1995 ▸)
*T*_min_, *T*_max_	0.869, 0.983
No. of measured, independent and observed [*I* > 2σ(*I*)] reflections	5289, 1345, 1105
*R*_int_	0.042
(sin θ/λ)_max_ (Å^−1^)	0.617

Refinement	
*R*[*F* ^2^ > 2σ(*F* ^2^)], *wR*(*F* ^2^), *S*	0.031, 0.074, 1.05
No. of reflections	1345
No. of parameters	119
H-atom treatment	H atoms treated by a mixture of independent and constrained refinement
Δρ_max_, Δρ_min_ (e Å^−3^)	0.27, −0.28

Computer programs: *COLLECT* (Nonius, 1998 ▸), *HKL* *DENZO* and *SCALEPACK* (Otwinowski & Minor, 1997 ▸), *SORTAV* (Blessing, 1995 ▸), *SHELXS97* (Sheldrick, 2008 ▸), *SHELXL2014/7* (Sheldrick, 2015 ▸), *ORTEP-3 for Windows* (Farrugia, 2012 ▸), *ATOMS* (Shape Software, 2007 ▸) and *publCIF* (Westrip, 2010 ▸).

## supplementary crystallographic information

### Crystal data

**Table:**

[Mn(C_7_H_7_N_2_O_2_)_2_]	*F*(000) = 366
*M**_r_* = 357.23	*D*_x_ = 1.724 Mg m^−^^3^
Monoclinic, *P*2_1_/*n*	Mo *K*α radiation, λ = 0.71073 Å
*a* = 11.5171 (5) Å	Cell parameters from 5315 reflections
*b* = 4.4212 (2) Å	θ = 1.0–26.0°
*c* = 13.9752 (7) Å	µ = 0.99 mm^−^^1^
β = 104.698 (3)°	*T* = 180 K
*V* = 688.32 (6) Å^3^	Slab, colourless
*Z* = 2	0.23 × 0.10 × 0.05 mm

### Data collection

**Table:**

Nonius KappaCCD diffractometer	1105 reflections with *I* > 2σ(*I*)
ω and φ scans	*R*_int_ = 0.042
Absorption correction: multi-scan (*SORTAV*; Blessing, 1995)	θ_max_ = 26.0°, θ_min_ = 2.1°
*T*_min_ = 0.869, *T*_max_ = 0.983	*h* = −14→13
5289 measured reflections	*k* = −5→5
1345 independent reflections	*l* = −17→17

### Refinement

**Table:**

Refinement on *F*^2^	Hydrogen site location: mixed
Least-squares matrix: full	H atoms treated by a mixture of independent and constrained refinement
*R*[*F*^2^ > 2σ(*F*^2^)] = 0.031	*w* = 1/[σ^2^(*F*_o_^2^) + (0.0282*P*)^2^ + 0.509*P*] where *P* = (*F*_o_^2^ + 2*F*_c_^2^)/3
*wR*(*F*^2^) = 0.074	(Δ/σ)_max_ < 0.001
*S* = 1.05	Δρ_max_ = 0.27 e Å^−^^3^
1345 reflections	Δρ_min_ = −0.28 e Å^−^^3^
119 parameters	Extinction correction: SHELXL2018/3 (Sheldrick 2015), Fc^*^=kFc[1+0.001xFc^2^λ^3^/sin(2θ)]^-1/4^
0 restraints	Extinction coefficient: 0.012 (2)
Primary atom site location: structure-invariant direct methods	

### Special details

**Table:**

Geometry. All esds (except the esd in the dihedral angle between two l.s. planes) are estimated using the full covariance matrix. The cell esds are taken into account individually in the estimation of esds in distances, angles and torsion angles; correlations between esds in cell parameters are only used when they are defined by crystal symmetry. An approximate (isotropic) treatment of cell esds is used for estimating esds involving l.s. planes.

### Fractional atomic coordinates and isotropic or equivalent isotropic displacement parameters (Å^2^)

**Table:**

	*x*	*y*	*z*	*U*_iso_*/*U*_eq_	
Mn1	0.000000	0.500000	0.000000	0.01883 (17)	
C1	0.32186 (18)	−0.0488 (5)	0.11717 (15)	0.0195 (5)	
C2	0.36414 (18)	0.1055 (5)	0.20598 (15)	0.0198 (5)	
H2	0.312438	0.241153	0.228031	0.024*	
C3	0.48037 (18)	0.0642 (5)	0.26264 (15)	0.0192 (5)	
C4	0.55799 (18)	−0.1335 (5)	0.23063 (16)	0.0204 (5)	
C5	0.51634 (19)	−0.2802 (5)	0.13985 (17)	0.0254 (5)	
H5	0.568982	−0.407707	0.115733	0.030*	
C6	0.39925 (19)	−0.2419 (5)	0.08470 (16)	0.0242 (5)	
H6	0.371484	−0.348284	0.024144	0.029*	
C7	0.19402 (18)	−0.0122 (5)	0.06079 (14)	0.0181 (4)	
N1	0.52161 (17)	0.2169 (4)	0.35541 (13)	0.0205 (4)	
H1N	0.482 (2)	0.387 (6)	0.3585 (18)	0.025*	
H2N	0.596 (2)	0.262 (6)	0.3646 (18)	0.025*	
N2	0.67763 (16)	−0.1663 (5)	0.28747 (15)	0.0229 (4)	
H3N	0.716 (2)	−0.306 (6)	0.2669 (18)	0.028*	
H4N	0.681 (2)	−0.190 (6)	0.350 (2)	0.028*	
O1	0.13370 (12)	0.1975 (3)	0.08586 (11)	0.0224 (4)	
O2	0.15011 (13)	−0.1998 (3)	−0.00841 (11)	0.0229 (4)	

### Atomic displacement parameters (Å^2^)

**Table:**

	*U*^11^	*U*^22^	*U*^33^	*U*^12^	*U*^13^	*U*^23^
Mn1	0.0190 (3)	0.0173 (3)	0.0187 (3)	0.00075 (19)	0.00188 (18)	0.00021 (19)
C1	0.0194 (10)	0.0188 (12)	0.0196 (10)	−0.0010 (8)	0.0034 (9)	0.0023 (8)
C2	0.0198 (10)	0.0176 (11)	0.0225 (11)	0.0022 (8)	0.0064 (9)	0.0021 (9)
C3	0.0198 (10)	0.0200 (12)	0.0171 (10)	−0.0027 (8)	0.0033 (8)	0.0013 (8)
C4	0.0165 (10)	0.0203 (11)	0.0238 (11)	−0.0015 (9)	0.0041 (9)	0.0039 (9)
C5	0.0233 (11)	0.0276 (13)	0.0265 (12)	0.0059 (10)	0.0088 (9)	−0.0022 (10)
C6	0.0258 (11)	0.0256 (12)	0.0201 (11)	0.0009 (10)	0.0036 (9)	−0.0028 (9)
C7	0.0198 (10)	0.0176 (10)	0.0168 (10)	−0.0030 (9)	0.0044 (8)	0.0042 (9)
N1	0.0189 (9)	0.0182 (10)	0.0230 (9)	−0.0018 (8)	0.0027 (8)	−0.0021 (8)
N2	0.0185 (9)	0.0273 (11)	0.0232 (10)	0.0037 (8)	0.0058 (8)	0.0013 (9)
O1	0.0208 (8)	0.0215 (8)	0.0234 (8)	0.0047 (6)	0.0026 (6)	0.0013 (6)
O2	0.0227 (8)	0.0232 (8)	0.0215 (8)	−0.0022 (6)	0.0031 (6)	−0.0017 (7)

### Geometric parameters (Å, º)

**Table:**

Mn1—O1	2.1591 (14)	C3—N1	1.432 (3)
Mn1—O1^i^	2.1591 (14)	C4—C5	1.397 (3)
Mn1—O2^ii^	2.2062 (15)	C4—N2	1.413 (3)
Mn1—O2^iii^	2.2062 (15)	C5—C6	1.383 (3)
Mn1—N1^iv^	2.3065 (19)	C5—H5	0.9500
Mn1—N1^v^	2.3065 (19)	C6—H6	0.9500
C1—C6	1.391 (3)	C7—O1	1.261 (3)
C1—C2	1.392 (3)	C7—O2	1.277 (3)
C1—C7	1.492 (3)	N1—H1N	0.88 (3)
C2—C3	1.384 (3)	N1—H2N	0.86 (3)
C2—H2	0.9500	N2—H3N	0.85 (3)
C3—C4	1.402 (3)	N2—H4N	0.88 (3)
			
O1—Mn1—O1^i^	180.0	C5—C4—C3	118.65 (19)
O1—Mn1—O2^ii^	93.20 (6)	C5—C4—N2	121.6 (2)
O1^i^—Mn1—O2^ii^	86.80 (6)	C3—C4—N2	119.64 (19)
O1—Mn1—O2^iii^	86.80 (6)	C6—C5—C4	120.7 (2)
O1^i^—Mn1—O2^iii^	93.20 (6)	C6—C5—H5	119.7
O2^ii^—Mn1—O2^iii^	180.0	C4—C5—H5	119.7
O1—Mn1—N1^iv^	90.52 (6)	C5—C6—C1	120.6 (2)
O1^i^—Mn1—N1^iv^	89.48 (6)	C5—C6—H6	119.7
O2^ii^—Mn1—N1^iv^	93.25 (6)	C1—C6—H6	119.7
O2^iii^—Mn1—N1^iv^	86.75 (6)	O1—C7—O2	123.23 (18)
O1—Mn1—N1^v^	89.48 (6)	O1—C7—C1	118.20 (18)
O1^i^—Mn1—N1^v^	90.52 (6)	O2—C7—C1	118.53 (19)
O2^ii^—Mn1—N1^v^	86.75 (6)	C3—N1—Mn1^vi^	120.83 (14)
O2^iii^—Mn1—N1^v^	93.25 (6)	C3—N1—H1N	112.9 (16)
N1^iv^—Mn1—N1^v^	180.0	Mn1^vi^—N1—H1N	98.0 (16)
C6—C1—C2	118.93 (19)	C3—N1—H2N	109.6 (17)
C6—C1—C7	121.50 (18)	Mn1^vi^—N1—H2N	107.0 (17)
C2—C1—C7	119.54 (19)	H1N—N1—H2N	107 (2)
C3—C2—C1	121.0 (2)	C4—N2—H3N	113.5 (17)
C3—C2—H2	119.5	C4—N2—H4N	111.1 (16)
C1—C2—H2	119.5	H3N—N2—H4N	111 (2)
C2—C3—C4	120.14 (19)	C7—O1—Mn1	131.83 (13)
C2—C3—N1	120.44 (19)	C7—O2—Mn1^vii^	121.03 (13)
C4—C3—N1	119.41 (18)		
			
C6—C1—C2—C3	−1.4 (3)	C7—C1—C6—C5	−177.8 (2)
C7—C1—C2—C3	176.57 (19)	C6—C1—C7—O1	−169.7 (2)
C1—C2—C3—C4	0.5 (3)	C2—C1—C7—O1	12.4 (3)
C1—C2—C3—N1	−178.0 (2)	C6—C1—C7—O2	12.3 (3)
C2—C3—C4—C5	1.6 (3)	C2—C1—C7—O2	−165.55 (19)
N1—C3—C4—C5	−179.9 (2)	C2—C3—N1—Mn1^vi^	89.4 (2)
C2—C3—C4—N2	177.9 (2)	C4—C3—N1—Mn1^vi^	−89.1 (2)
N1—C3—C4—N2	−3.5 (3)	O2—C7—O1—Mn1	−40.5 (3)
C3—C4—C5—C6	−2.9 (3)	C1—C7—O1—Mn1	141.59 (15)
N2—C4—C5—C6	−179.1 (2)	O1—C7—O2—Mn1^vii^	−60.8 (2)
C4—C5—C6—C1	2.0 (3)	C1—C7—O2—Mn1^vii^	117.02 (17)
C2—C1—C6—C5	0.1 (3)		

Symmetry codes: (i) −*x*, −*y*+1, −*z*; (ii) −*x*, −*y*, −*z*; (iii) *x*, *y*+1, *z*; (iv) *x*−1/2, −*y*+1/2, *z*−1/2; (v) −*x*+1/2, *y*+1/2, −*z*+1/2; (vi) −*x*+1/2, *y*−1/2, −*z*+1/2; (vii) *x*, *y*−1, *z*.

### Hydrogen-bond geometry (Å, º)

**Table:**

*D*—H···*A*	*D*—H	H···*A*	*D*···*A*	*D*—H···*A*
N1—H1*N*···O1^v^	0.88 (3)	2.19 (3)	3.022 (3)	156 (2)
N2—H3*N*···N2^viii^	0.85 (3)	2.26 (3)	3.106 (3)	178 (2)
N2—H4*N*···O2^ix^	0.88 (3)	2.15 (3)	3.008 (2)	167 (2)

Symmetry codes: (v) −*x*+1/2, *y*+1/2, −*z*+1/2; (viii) −*x*+3/2, *y*−1/2, −*z*+1/2; (ix) *x*+1/2, −*y*−1/2, *z*+1/2.
