# Supplementary material for: Hydro­thermal synthesis and crystal structure of poly[bis­(μ3-3,4-di­amino­benzoato)manganese], a layered coordination polymer
Source: Acta Crystallogr E Crystallogr Commun. 2020 May 22;76(Pt 6):909–13. doi: 10.1107/S2056989020006805 (PMC7273982; doi:10.1107/S2056989020006805)
Supplement: Supplementary file 3 [file e-76-00909-sup3.docx]

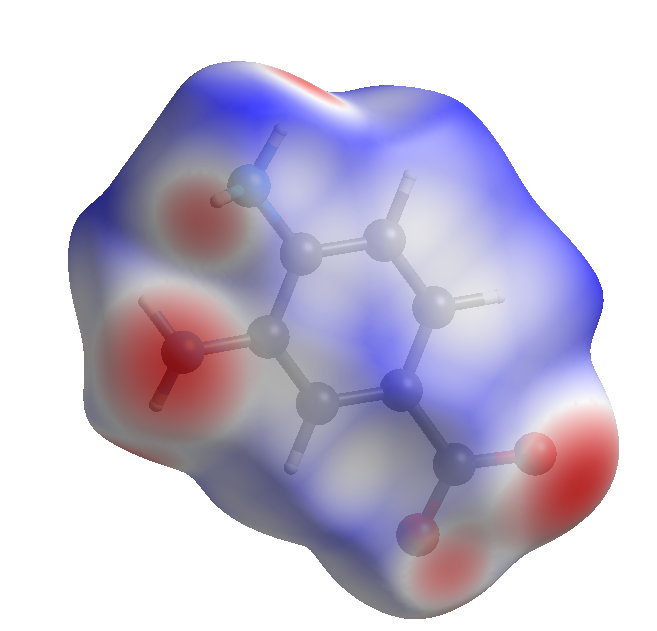


**Figure S1**. Hirshfeld surface of the C_7_H_7_N_2_O_2_^–^ anion in (I). The manganese cation was moved to a symmetry-equivalent site to generate this image and is not shown.
